# Combining QTL mapping with transcriptome and metabolome profiling reveals a possible role for ABA signaling in resistance against the cabbage whitefly in cabbage

**DOI:** 10.1371/journal.pone.0206103

**Published:** 2018-11-06

**Authors:** Colette Broekgaarden, Koen T. B. Pelgrom, Johan Bucher, Nicole M. van Dam, Katharine Grosser, Corné M. J. Pieterse, Martijn van Kaauwen, Greet Steenhuis, Roeland E. Voorrips, Martin de Vos, Ben Vosman, Anja Worrich, Saskia C. M. van Wees

**Affiliations:** 1 Plant-Microbe Interactions, Department of Biology, Science4Life, Utrecht University, Utrecht, the Netherlands; 2 Wageningen UR Plant Breeding, Wageningen University and Research Centre, Wageningen, the Netherlands; 3 Keygene N.V., Wageningen, the Netherlands; 4 German Centre for Integrative Biodiversity Research (iDiv) Halle-Jena-Leipzig, Leipzig, Germany; 5 Friedrich Schiller University Jena, Institute of Biodiversity, Jena, Germany; Chinese Academy of Agricultural Sciences, CHINA

## Abstract

Whiteflies are among the world’s most significant agricultural pests and chemical insecticides are extensively used to reduce crop damage to acceptable levels. However, nearly all insecticides pose a threat to the environment and alternative control methods, such as breeding of crop varieties that are inherently insect-resistant, are needed. Previously, a strong source of plant-age dependent resistance to the cabbage whitefly (*Aleyrodes proletella*) has been identified in the modern white cabbage (*Brassica oleracea* var. *capitata*) variety Rivera. However, nothing is known about the molecular mechanisms or the genes involved in this resistance. In the present study, a multidisciplinary approach combining transcriptome and metabolome profiling with genetic mapping was used to identify the molecular players of whitefly resistance in cabbage. Transcriptome profiles of young (susceptible) and older (resistant) Rivera plants were analyzed using RNA sequencing. While many genes involved in general processes were differentially expressed between both ages, several defense-related processes were overrepresented in the transcriptome profile of older plants. Hormone measurements revealed that jasmonic acid (JA) levels decreased upon whitefly infestation at both plant ages. Interestingly, abscisic acid (ABA) levels showed contrasting effects in response to whitefly infestation: ABA levels were reduced in young plants but induced in older plants upon whitefly feeding. Auxin levels were significantly lower in older plants compared with young plants, independent of whitefly presence, while glucosinolate levels were higher. Additionally, whitefly performance was monitored in an F_2_ population derived from a cross between Rivera and the susceptible white cabbage variety Christmas Drumhead. Significant QTL intervals were mapped on chromosome 2 and 9 for oviposition rate and whitefly adult survival, respectively. Several genes that were higher expressed in older plants and located in the identified QTL intervals were orthologous to Arabidopsis genes that have been related to ABA signaling, suggesting a role for ABA in the regulation of resistance towards whiteflies. Our results show that combining different omics approaches is a useful strategy to identify candidate genes underlying insect resistance.

## Introduction

Whiteflies are serious pests that cause substantial losses to a large number of agricultural crops worldwide. Using their stylet, whiteflies find their way through the host plant tissue to access the vascular system and feed for prolonged periods of time from the phloem sieve elements. Damage is not only attributed to direct feeding, but also to indirect effects of honeydew secretion facilitating the growth of mold and transmission of pathogenic viruses. At present, farmers use multiple applications of chemical insecticides to reduce whitefly populations to acceptable levels but this leads to the development of resistance by whiteflies and mortality of beneficial insects. A more desirable and environmental friendly alternative is the exploitation of the plant’s inherent immune system [1,2].

The plant defense system is complex and characterized by a network of pathways in which antagonistic and synergistic interactions are considered important to fine tune the activation of specific defenses. The phytohormone jasmonic acid (JA) is generally considered the most important player in the battle against herbivorous insects [3,4]. Ethylene (ET) and abscisic acid (ABA) are believed to positively fine tune JA-mediated defenses against insects [5,6], while salicylic acid (SA) and auxin (IAA) are known to be negative regulators of JA-related plant responses [7]. JA signaling mediates the accumulation of glucosinolates, which are major defensive secondary metabolites of Brassicaceous plants. The different types of glucosinolates often differentially affect insect performance, depending on the insect encountered. While aliphatic glucosinolates mainly affect the performance of chewing insects [8], the indole glucosinolates are usually the ones reducing the performance of phloem feeding insects [9–11].

A thorough understanding of the interaction between plants and whiteflies, including the molecular mechanisms underlying plant defense, is essential for the development of resistant varieties. Plant responses towards whitefly infestation seems to vary depending on the species involved [12]. In cotton, for example, infestation by the silverleaf whitefly (*Bemisia tabaci*) results in enhanced expression of defense genes leading to the production of defensive secondary metabolites [13]. Conversely, in Arabidopsis (*Arabidopsis thaliana*) the silverleaf whitefly manipulates the plant’s response for its own benefit by inducing SA signaling to suppress effectual JA defenses [14]. Several sources of host plant resistance towards different whitefly species have been identified that either negatively affect host plant selection [15] or reduce whitefly survival and/or reproduction [16,17]. Quantitative Trait Loci (QTL) analysis has been successfully used to identify genomic regions that contribute to resistance [18–20]. For example, a region at the top of chromosome 5 of the wild tomato species *Solanum habrochaites* is associated with reduced oviposition by the silverleaf whitefly [21]. Currently, only one NBS-LRR type resistance (R) gene conferring resistance to whiteflies has been identified, i.e., the *Mi-1*.*2* gene from tomato suppresses population buildup of the silverleaf whitefly [22].

The cabbage whitefly, *Aleyrodes proletella*, is a pest on various *Brassica* crops and reduces marketability of especially Kale, Brussels sprouts and Savoy cabbage. Previously, a strong source of resistance against this whitefly has been identified in the modern white cabbage (*Brassica oleracea* var. *capitata*) variety Rivera [23]. The observed resistance is not affected by environmental factors but highly depends on plant age: all whitefly life stages perform well on 6-weeks-old plants whereas adults die quickly and hardly lay any eggs on 12-weeks-old plants. Moreover, previous results indicate that morphological differences between plant ages are not associated with the observed resistance [23]. In the present study, we characterize the molecular players involved in resistance against the cabbage whitefly in Rivera. Our main objectives were to (1) evaluate in more detail the development of whitefly resistance during plant development, (2) analyze and compare the transcriptional and metabolic profiles of young (susceptible) and older (resistant) plants in the absence and presence of whiteflies, (3) study the genetics of resistance using a QTL mapping approach, and (4) identify candidate genes involved in the resistance by combining results of the QTL mapping with transcriptome and metabolome profiling.

## Materials & methods

### Plant material and growth conditions

Seeds from the white cabbage (*Brassica oleracea* var. *capitata*) F_1_ hybrid variety Rivera were obtained from Bejo Zaden B.V. (Warmenhuizen, the Netherlands). An F_2_ mapping population was developed by selfing a single F_1_ plant derived from the controlled pollination of the whitefly-susceptible, open-pollinated white cabbage landrace Christmas Drumhead (obtained from the Centre of Genetic Resources, Wageningen, the Netherlands) by whitefly-resistant variety Rivera [23,24].

For all experiments, seeds were germinated in potting compost (Lentse Potgrond, Lent, Netherlands) in a greenhouse compartment at 20±2°C with an L16:D8 photoperiod and 40–70% RH. Plants for the field experiment were individually transferred to peat soil blocks 1 week after germination and transplanted into the field after an additional 4 weeks. For greenhouse experiments, plants were transferred to 19-cm pots 4 weeks after germination and grown until the desired age under the above mentioned greenhouse conditions. Plants were watered every other day, fertilized with 2.5 mg l^-1^ Kristalon Blauw (N-P-K-MgO, 19-6-20-3; Hydro Agri, Rotterdam, the Netherlands) every 3 weeks and received no chemical control for pests and diseases.

### Insects

The population of cabbage whitefly, *Aleyrodes proletella* (Hemiptera: Aleyrodidae), originated from adults collected in 2008 from a white cabbage field near Wageningen, Netherlands (51^0^57’N, 5^0^38’E) [23]. This population was maintained on Brussels sprouts (*B*. *oleracea* var. *gemmifera* cv. Cyrus) in a climate chamber at 20±2°C with an L16:D8 photoperiod and 40–60% RH. Whiteflies were reared under conditions in which there was always sufficient foliage for feeding and oviposition. For all experiments, adult female whiteflies of assorted ages were randomly collected from the rearing using an aspirator. Whiteflies were briefly (<30 min) anaesthetized with a gas mixture (N_2_:H_2_:CO_2_ 80:10:10; Linde Gas Benelux B.V., Schiedam, the Netherlands) to enable selection and transfer of females.

### Whitefly performance on greenhouse-grown plants of different ages

Plants received 2 clip cages (diameter 2 cm, height 1.2 cm), each containing 5 whitefly females, on the abaxial surface of 2 young, fully expanded leaves of 5 plants per age to obtain 5 biological replicates. Whiteflies were allowed to feed and oviposit for 7 days during which adult survival (n^o^ females alive / total n^o^ females) was monitored daily. The number of eggs was counted after these 7 days to be able to calculate the oviposition rate represented by the daily number of eggs laid per female (eggs·female^-1^·day^-1^). Values were averaged per plant and arcsine-square-root-transformed for survival or log_10_(*x* + 0.1)-transformed for oviposition rate to normalize the distribution of the residuals. Survival curves of whiteflies were analyzed using general linear model (GLM) repeated measures ANOVA followed by LSD tests. Day was considered a within-subjects factor and plant age a between-subjects factor. Comparisons between the different plant ages for oviposition rate were made using ANOVA followed by LSD tests. Differences were considered significant when *P* < 0.05.

### Gene expression analyses

#### Whitefly infestation

**Fig 1:**
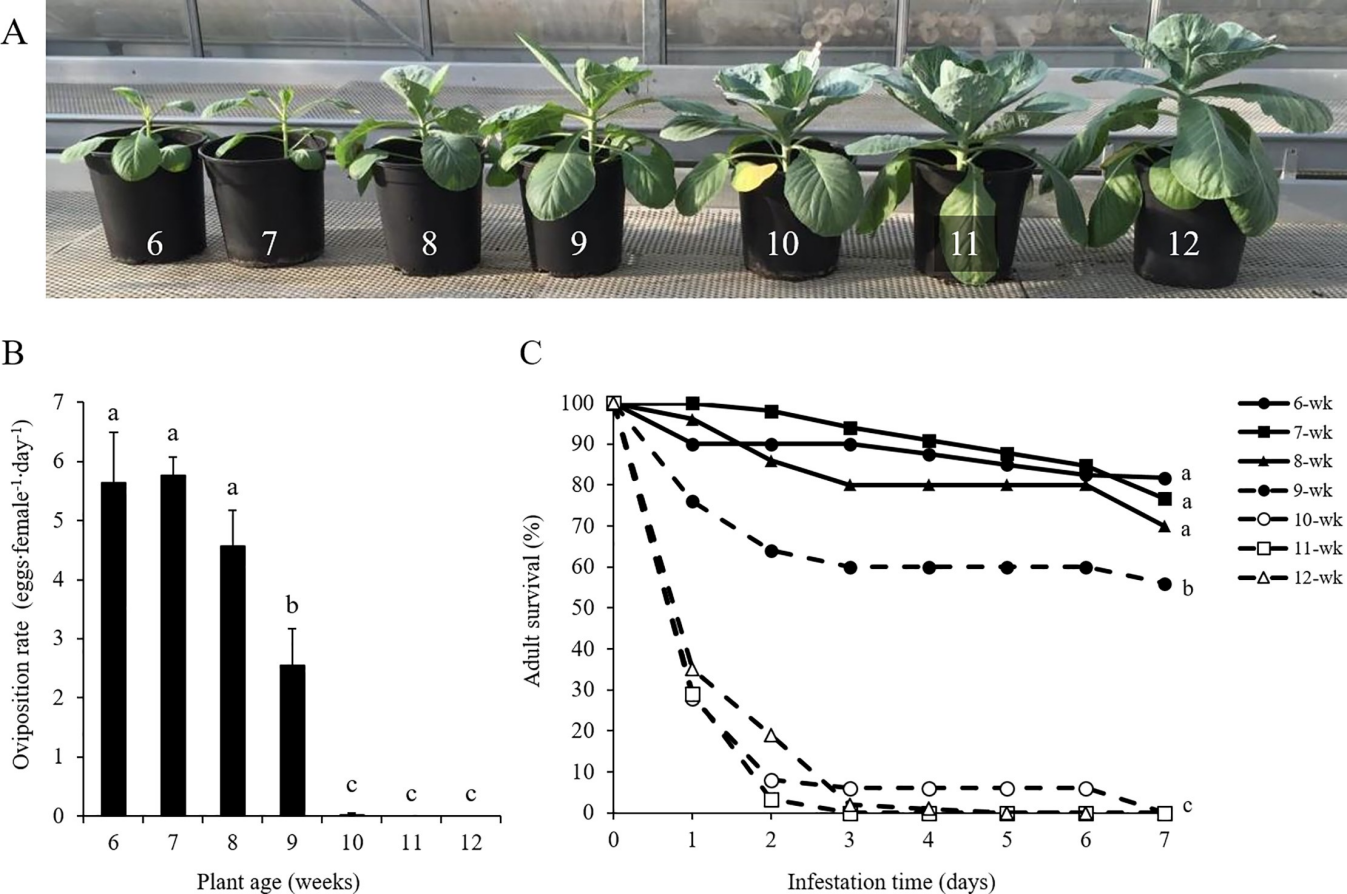
Performance of *Aleyrodes proletella* on *Brassica oleracea* cv. Rivera of different ages. (A) Appearance of plants used for the experiment, ranging from 6- to 12-weeks-old. Plant ages are indicated with numbers on the pots. (B) Oviposition rate (eggs·female^-1^·day^-1^; mean + SE) and (C) daily survival (%) of whitefly females on leaves of the different plant ages. Values represent means of 5 plants per age (2 clip cages enclosing 5 females each per plant). Lines/bars marked with different letters indicate significant differences between plant ages (*p* < 0.05).

Seven- and 13-weeks-old Rivera plants (Fig 1A) were infested with female whitefly adults of assorted ages. Individual plants received 2 clip cages (diameter 2 cm, height 1.2 cm), each containing 20 female whiteflies, on the abaxial surface of the 2 youngest, fully expanded leaves. Control plants received 2 empty clip cages on similar locations as the induction plants. After 4 hours, leaf discs (diameter 2.3 cm) were collected inside the clip cages and leaf discs of 3 plants were pooled to obtain a biological replicate. The same procedure was repeated on 2 other sets of plants for 2 additional biological replicates. A total of 12 samples were collected, i.e., 3 biological replicates for each age and treatment. Samples were immediately frozen in liquid nitrogen and stored at −80°C until use. None of the whiteflies had escaped from the cages during the infestation period.

#### Whitefly performance

One of the infested leaves of the induction plants (described in the previous paragraph) received an additional clip cage containing 5 female whiteflies to make sure the sampled plants have the expected phenotype, i.e., 7-weeks-old plants are susceptible and 13-weeks-old plants are fully resistant. The females were allowed to feed and oviposit for 4 days after which the number of whiteflies (dead and alive) and eggs were counted. Adult survival (n^o^ females alive / total n^o^ females) and oviposition rate (eggs·female^-1^·day^-1^) were calculated per clip cage and subsequently averaged for the pools of 3 plants.

#### RNA extraction, cDNA library preparation and Illumina sequencing

Leaf samples were ground in liquid nitrogen and total RNA was extracted using the RNeasy Plant Mini kit (Qiagen, Hilden, Germany) following the manufacturer’s protocol. Total RNA was quantified by Nanodrop 1000 and analyzed with the Agilent 2100 Bioanalyzer (Agilent Technologies, Santa Clara, CA, USA) to confirm its integrity before sequencing. RNA sequencing (RNA-seq) library construction and sequencing was done at the Utrecht Sequencing Facility (Utrecht, the Netherlands). Libraries were constructed using the TruSeq Stranded mRNA polyA Sample Preparation kit (Illumina, San Diego, USA) and thereafter sequenced on an Illumina NextSeq500 platform using a paired-end 150-base pair high output run [25]. The raw RNA-seq read data are deposited in the Short Read Archive (http://www.ncbi.nlm.nih.gov/bioproject/PRJNA490257).

#### RNA-seq data analysis

Quality control, processing of the reads and alignment to the *Brassica oleracea* reference genome [26] was done using Kallisto [27]. Filtering, normalization and differential expression calls were performed using the R package EdgeR [28]. Differentially expressed genes (DEGs) were identified by a 2-way ANOVA with the factors age (7- or 13-weeks-old) and treatment (whitefly infested or control) on log-transformed normalized read counts. A Holm correction was used to correct *P*-values for multiple testing and genes were considered differentially expressed when this corrected *P* < 0.05. Annotations based on the Arabidopsis genome [26] were used to predict the function of orthologous *B*. *oleracea* unigenes. Gene ontology (GO) enrichment analysis was performed using PANTHER v13.1 [29] on biological process terms from Arabidopsis. GO terms with a False Discovery Rate (FDR) corrected *P* < 0.05 were considered.

### Hormone and glucosinolate quantification

The grounded material used for RNA-seq was also used for hormone measurements. Frozen material was used to analyze the plant hormones 12-oxo-phytodienoic acid (OPDA), jasmonic acid (JA), JA-isoleucine conjugate (JA-Ile), salicylic acid (SA), abscisic acid (ABA) and auxin (IAA) by UPLC-ESI-MS/MS Synapt G2-S HDMS according to the protocol in Papadopoulou et al. [30]. Samples for glucosinolate measurements were freeze-dried, extracted according to Grosser and van Dam [31] and subsequently analyzed by HPLC. Concentrations were calculated over the amount of fresh weight (for hormones) or dry weight (for glucosinolates). Differences in concentrations were determined using 2-way ANOVA on log_10_-transformed data with age and treatment as factors. Differences were considered significant when *P* < 0.05.

### Phenotyping the F_2_ population

In 2011, a field experiment was performed at a site nearby Wageningen, the Netherlands (51^0^57’N, 5^0^38’E; clay soil, conventionally managed). Five-weeks-old plants were transplanted into the field with a plant distance of 70 x 70 cm. In total, 179 F_2_ plants and 10 plants of each parent were randomized and divided over 2 blocks. The blocks were surrounded by a row of remaining F_2_ plants to minimize edge effects. A 6-m strip sown with *Lolium* and *Poa* grasses separated the blocks. No insecticides or fungicides were applied and weeds were manually removed from the field.

When plants were 12 weeks old, each plant received 4 clip cages (diameter 2 cm, height 1.2 cm), containing 5 female whiteflies on the abaxial surface of 4 young, fully expanded leaves. Due to practical reasons and weather conditions, plants were infested over a 4-day period in which individual plants received 1 clip cage a day. Whitefly females were allowed to feed and oviposit for 5–7 days (depending on the rainfall) after which the number of whiteflies (dead and alive) and eggs were counted. Several whiteflies were able to escape from the clip cages due to irregular leaf surface. Data from clip cages with less than 4 whiteflies (alive + dead) were removed from the analysis, as well as data from plants with less than 2 remaining clip cage observations. After this filtering step, further analysis was done with data of 133 F_2_, 7 Rivera and 9 Christmas Drumhead plants. Adult survival (n^o^ females alive / total n^o^ females) and oviposition rate (eggs·female^-1^·day^-1^) were calculated per clip cage and averaged per plant. The individual plant observations were transformed to normalize the distribution of the residuals using arcsine-square root-transformation for adult survival and a log_10_ (*x* + 1)-transformation for oviposition rate. Pearson correlation tests were used to determine the relatedness between whitefly performance parameters.

### DNA extraction and genotyping

Samples of young leaves from the parents and F_2_ plants were collected and stored at -80 ^o^C until DNA extraction. Samples were ground using the Retsch Mixer Mill MM301 (Retsch GmbH, Haan, Germany) according to the manufacturer’s manual and DNA was extracted according to Fulton et al. [32]. DNA quantity and quality were determined with NanoDrop 1000 V.3.7 (Thermo Fisher Scientific Inc).

DNA solutions (5 ng·μl^-1^) were prepared for genotyping single nucleotide polymorphism (SNP) by LGC KASPar technology carried out by dr. van Haeringen laboratorium B.V., Wageningen, the Netherlands. SNP markers were derived from a pool of 25 F_2_ plants, as leaf material of the original crossing parents and F_1_ plant was no longer available. In total, 187,669,310 read-pairs were sequenced using the HiSeq2000 Illumina platform. The overlapping pairs were merged using Flash with default settings [33]. This yielded 57,859,297 merged and 129,810,013 unmerged reads that were quality trimmed using ConDeTri with default settings [34]. After trimming, a *de novo* assembly was performed with SOAPdenovo (settings: k-mer size 41, insert length 225 bp, otherwise default) [35] in which 998,228 scaffolds & singletons were assembled. After removing fragments shorter than 400 bp, 236,398 sequences remained, with an N50 of 1546 bp. Using the assembled fragments >400 bp as a reference, the trimmed reads were mapped with Bowtie2 with default settings [36] and QualitySNP was used to retrieve SNP markers from the resulting sequence alignment file (sam) [37,38]. The minimum number of reads supporting a SNP was set to 5 and the threshold for total read coverage was set to 30%. The maximum number of haplotypes in each contig was set to 2. The 100-bp flanking region on each side of the SNP had to be free of other polymorphisms. Taking into account all these criteria, we identified 62,868 potential SNPs. To identify sequences having a high similarity with other loci, the flanking sequence (2 x100 bp) of each SNP was blasted against the assembled contigs, the *Brassica rapa* v1.2 genome sequence (e-value threshold 1e-20) and the *B*. *oleracea* genome sequence [39]. 45,960 SNP markers were then filtered having only 1 unique blast-hit on the scaffolds of the assembly and 1 unique blast-hit at the *Brassica* genome sequences. 150 SNP markers, evenly distributed across the genome, were selected for genotyping. Before creating a genetic linkage map we removed F_2_ plants and SNP markers with more than 10% missing data points.

### QTL analysis

#### Heritability

Broad-sense heritability of each trait (H^2^) was calculated as H^2^ = V_g_ / VF_2_, with V_g_ = VF_2_ –((VP1 + VP2)/2), where VF_2_ represents the variance among F_2_ plants, VP1 the variance among Christmas Drumhead plants, and VP2 the variance among Rivera plants.

#### Construction of linkage map

JoinMap 4.1 [40] was used to create a genetic linkage map, using the regression algorithm and Kosambi map function with a recombination frequency less than 0.4 and an experimental logarithm of odds (LOD) significance threshold > 1.0. Five F_2_ plants with identical marker genotypes and 6 SNP markers that showed identical segregation patterns with other markers were excluded before constructing a genetic linkage map. One SNP marker (C50BS11007597) was removed due to insufficient linkage with other markers. MapChart 2.2 was used to visualize the genetic map [41].

#### QTL mapping

Associations between markers and traits were detected by interval mapping using MapQTL 6 [42]. QTL analysis for morphological traits was performed using 171 F_2_ plants whereas QTL analysis for whitefly performance was performed using a subset of 125 F_2_ plants due to filtering steps in the phenotypic and genotypic analyses (see above). A permutation test (1000 iterations) was performed to determine the LOD threshold for each trait with a genome-wide confidence level of 0.05. The 1-LOD and 2-LOD support intervals were determined per trait. Multiple-QTL model (MQM) mapping, in which we assigned the markers with the highest LOD score as co-factor, did not discover any additional QTLs.

## Results

### Development of whitefly resistance during plant growth

To determine the development of resistance during plant growth, we monitored whitefly performance on greenhouse-grown plants of 6- to 12-weeks-old (Fig 1A). Oviposition rate on young plants, i.e. 6- to 8-weeks-old, varied between 4.5 and 5.8 eggs∙female^-1^∙day^-1^ (Fig 1B). On 9-weeks-old plants, oviposition rate was significantly lower than that on the younger plants, namely 2.6 eggs∙female^-1^∙day^-1^. From the age of 10 weeks onwards, plants became highly resistant. Oviposition rate was very close to zero on 10-weeks-old plants and no eggs at all were observed on 11- and 12-weeks-old plants (Fig 1B). Alike oviposition rate, daily monitoring the survival of whitefly adults revealed that 6-, 7- and 8-weeks-old plants were equally susceptible with 70–80% survival a week after infestation (Fig 1C). On 9-weeks-old plants, whitefly adults died significantly faster than on plants that were younger, but significantly slower than on plants that were older. More than 80% of the whiteflies placed on 10-, 11- and 12-weeks-old plants died within 2 days and after 7 days all whiteflies were dead, which was significantly faster than on all the younger plants (Fig 1C). These results indicate that young Rivera plants (6- to 8-weeks-old) are highly susceptible to whiteflies but become resistant at the age of 10 weeks with a short transition phase in between. When coupling the appearance of plants (Fig 1A) to the cabbage developmental scale [43], the transition from susceptible to resistance occurs more or less together with the start of head formation.

### Transcriptional changes during plant development

To evaluate transcriptional changes during plant growth, we performed transcriptional profiling (RNA-sequencing) on Rivera leaves of a susceptible age (7-weeks-old) and a resistant age (13-weeks-old) that were either infested by whiteflies for 4 hours or left uninfested. Whitefly performance was also monitored on the same plants to make sure that the plants showed the expected phenotype. Indeed, 7-weeks-old plants were all susceptible, with an average adult survival of > 60% and oviposition rate of > 4.5 eggs·female^-1^·day^-1^, whereas 13-weeks-old plants were all completely resistant, i.e. no adult survival and no to 0.1 eggs ·female^-1^·day^-1^ (S1 Table).

**Fig 2:**
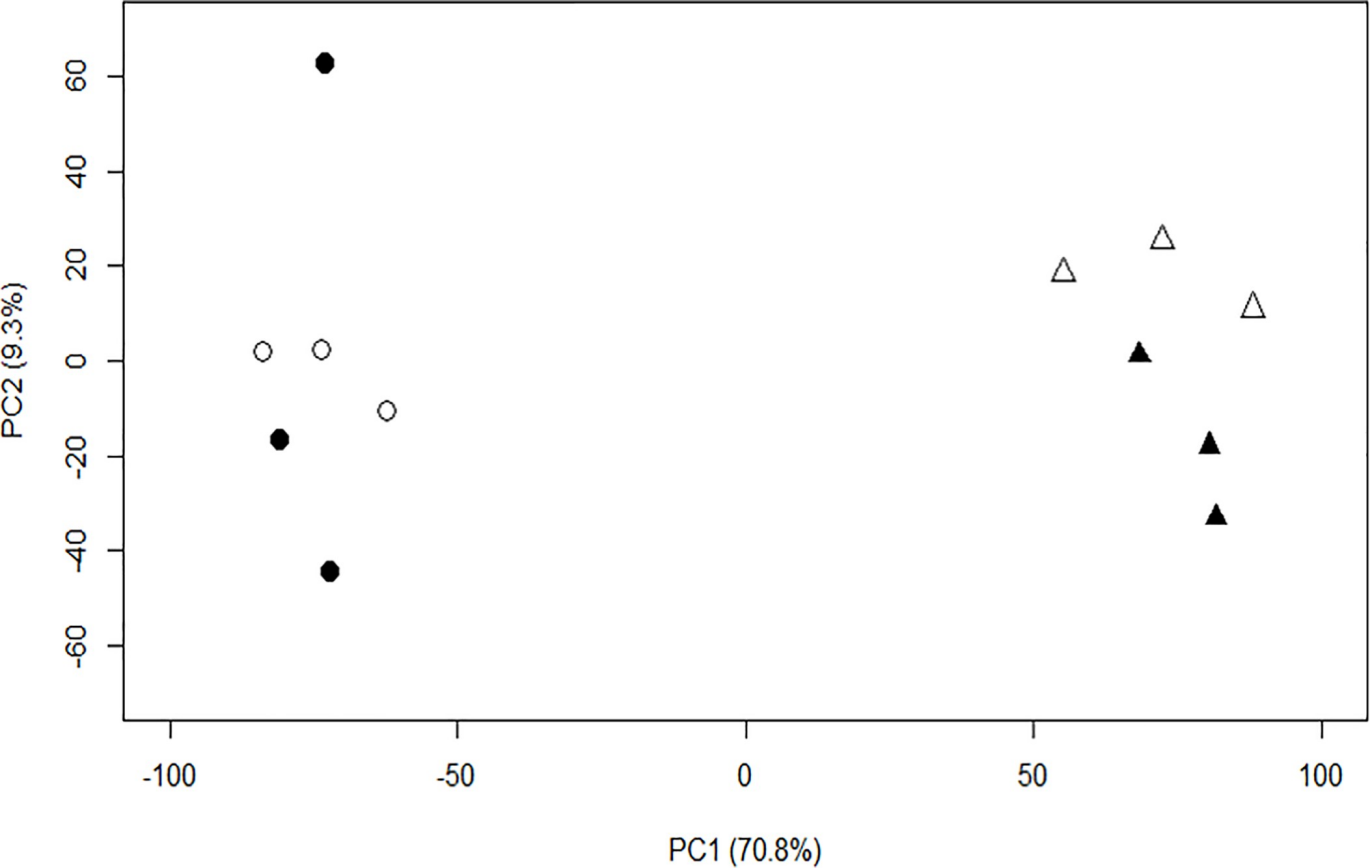
Principal component analysis (PCA) of gene expression levels in young and older Rivera plants. Circles represent young (7-weeks-old) plants while older (13-weeks-old) plants are represented by triangles. Plants were either untreated (open symbols) or infested with *Aleyrodes proletella* for 4 hours (closed symbols). Score plot of the first 2 principal components (PC) with the explained variance in parentheses.

A principal component analysis (PCA) using all the mapped reads showed that transcriptional profiles were highly dependent on plant age. The first PC clearly separated the plant ages and explained 70.8% of the variation while the second PC, explaining 9.3% of the variation, separated control and whitefly-infested samples of the older plants but not of the young plants (Fig 2). Because most of the variation is explained by plant age and it is not known whether the observed resistance is whitefly-inducible or constitutive, we focused on the genes differentially expressed between the plant ages, either dependent or independent of whitefly presence. In total, 2043 genes were differentially expressed between 7- and 13-weeks-old plants (2-way ANOVA, Holm adjusted *P* < 0.05; S2 Table). Homology annotations based on the Arabidopsis genome [26] were used to predict the function of *B*. *oleracea* unigenes. A GO enrichment analysis using these homology annotations revealed that the 1317 differentially expressed genes (DEGs) with higher expression levels in young plants are mainly enriched for general processes, such as photosynthesis and primary metabolism (S3 Table). The 726 DEGs with higher expression levels in older plants are enriched for, amongst others, processes related to hormone signaling and secondary metabolite biosynthesis (S4 Table), including several well-known genes involved in plant defense against insects. For example, Bo5g086990 and Bo8g028850 are orthologues of Arabidopsis *MYC2*, a transcriptional regulator of JA-mediated defense signaling and crosstalk mediator between JA and other hormones [44] (S2 Table). Also the orthologue of Arabidopsis *MYC3* (Bo7g075710), a close homologue of *MYC2* that additively regulated defense against insects [45], was stronger expressed in older plants than in young plants. Both *MYC2* and *MYC3* have also been shown to regulate glucosinolate biosynthesis [8]. Additionally, Bo4g169370 is higher expression in older plants than in younger plants and is orthologous to Arabidopsis *GSTU4*, a gene involved in indole glucosinolate metabolism during immune responses [46] (S2 Table).

### Hormone and glucosinolate accumulation

**Fig 3:**
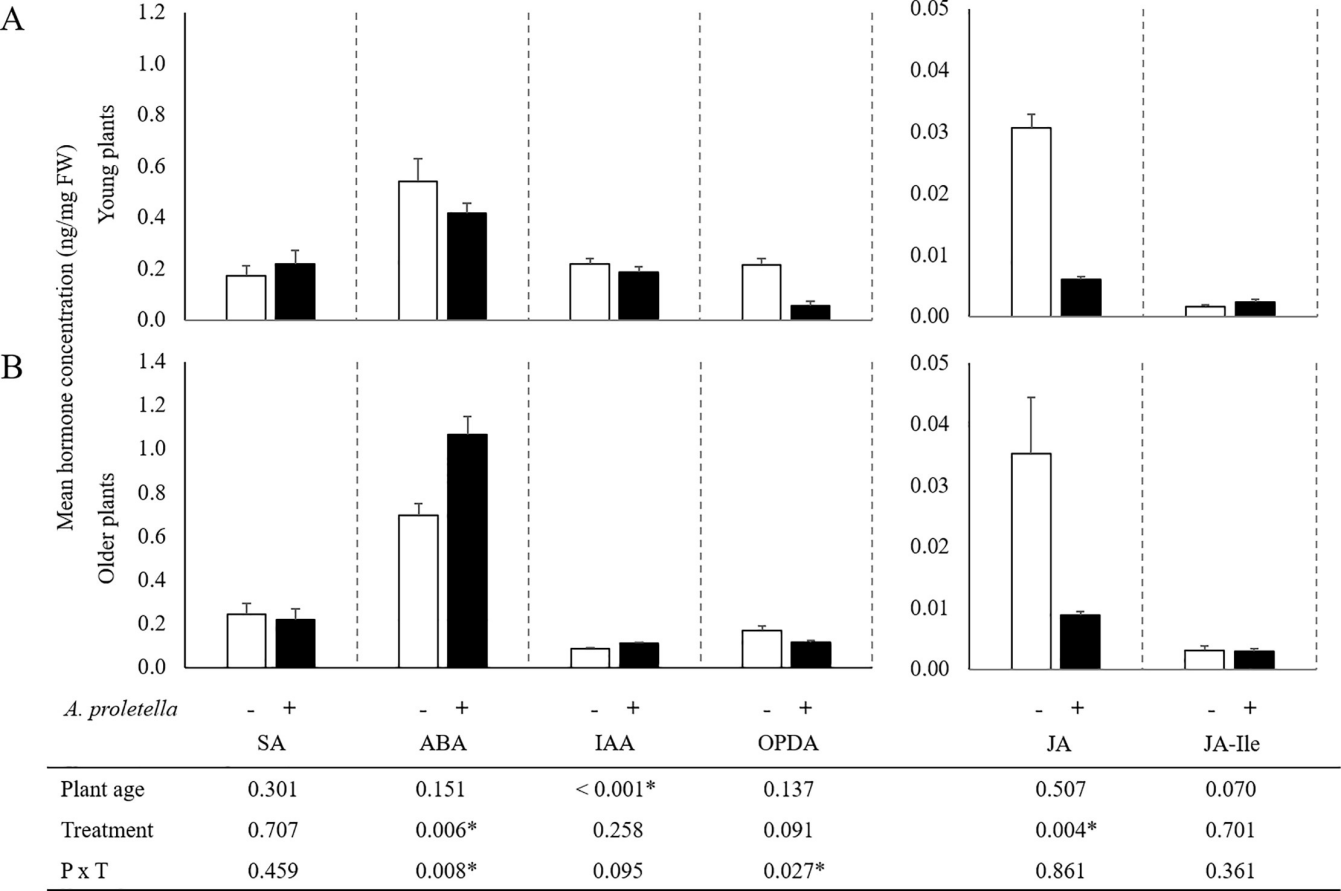
Effect of plant age and infestation by *A*. *proletella* on hormone accumulation in Rivera leaves. Concentrations in young (A) and older (B) plants of 12-oxo-phytodienoic acid (OPDA), jasmonic acid (JA), JA-isoleucine conjugate (JA-Ile), salicylic acid (SA), abscisic acid (ABA) and auxin (IAA) are presented as ng‧mg^-1^ fresh weight (mean + SE, n = 3). Control plants: white bars, whitefly-infested plants: black bars. Table at the bottom represents *P*-values from 2-way ANOVA analyses. Stars indicate significant effects (*P* < 0.05) for the individual factors or the factorial interaction.

Because the transcriptome data indicated a difference in hormone signaling and secondary metabolite biosynthesis between the plant ages, we also analyzed concentrations of defense-related hormones and glucosinolates in the same leaf samples. Auxin (IAA) concentrations were lower in older plants than in younger plants, independent of whitefly presence (Fig 3). Upon whitefly infestation, JA concentrations were significantly reduced at both plant ages. In young plants, also concentrations of the JA precursor OPDA were negatively affected by whitefly infestation. Interestingly, ABA levels were repressed by whitefly infestation in young plants but much stronger induced in older plants. SA and JA-Ille were not affected by plant age nor by whitefly presence (Fig 3).

**Fig 4:**
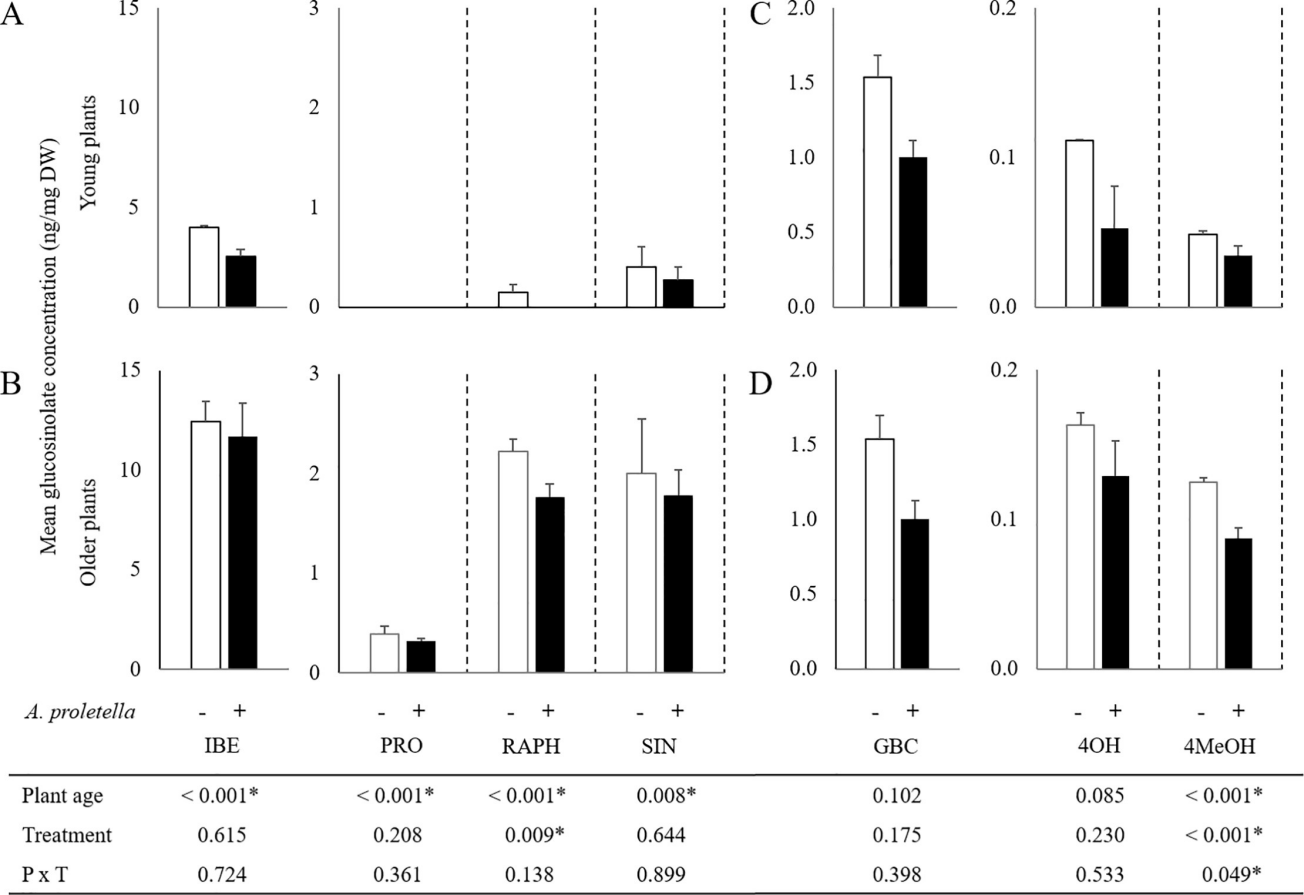
Effect of plant age and infestation by *A*. *proletella* on glucosinolate concentrations in Rivera leaves. Shown are the concentrations as ng·mg^-1^ dry weight (mean + SE) for the aliphatic (A,B) and indole (C,D) glucosinolates in young (A,C) and older (B,D) plants. IBE, Glucoiberin; PRO, Progoitrin; RAPH, Glucoraphanin; SIN, Sinigrin; 4OH, 4-Hydroxy-glucobrassicin; GBC, Glucobrassicin; 4MeOH, 4-Methoxy-glucobrassicin. Control plants: white bars, whitefly-infested plants: black bars. Table at the bottom represents *P*-values from 2-way ANOVA analyses. Stars indicate significant effects (*P* < 0.05) for the individual factors or the factorial interaction.

For all the detectable aliphatic glucosinolates, concentrations were higher in older plants than in younger plants and both plant ages responded similarly to whitefly infestation, i.e., no effect for glucoiberin (IBE), progoitrin (PRO) and sinigrin (SIN) levels and a reduction of glucoraphanin (RAPH) levels upon whitefly infestation (Fig 4). Among the detectable indole glucosinolates, only 4-methoxy-glucobrassicin (4MeOH) levels were affected by plant age as well as by whitefly infestation. Overall 4MeOH levels were slightly higher in older plants and repressed in whitefly-infested leaves compared with control leaves. This reduction was slightly stronger in older than in younger plants (Fig 4).

### Whitefly performance in an F_2_ population

**Fig 5:**
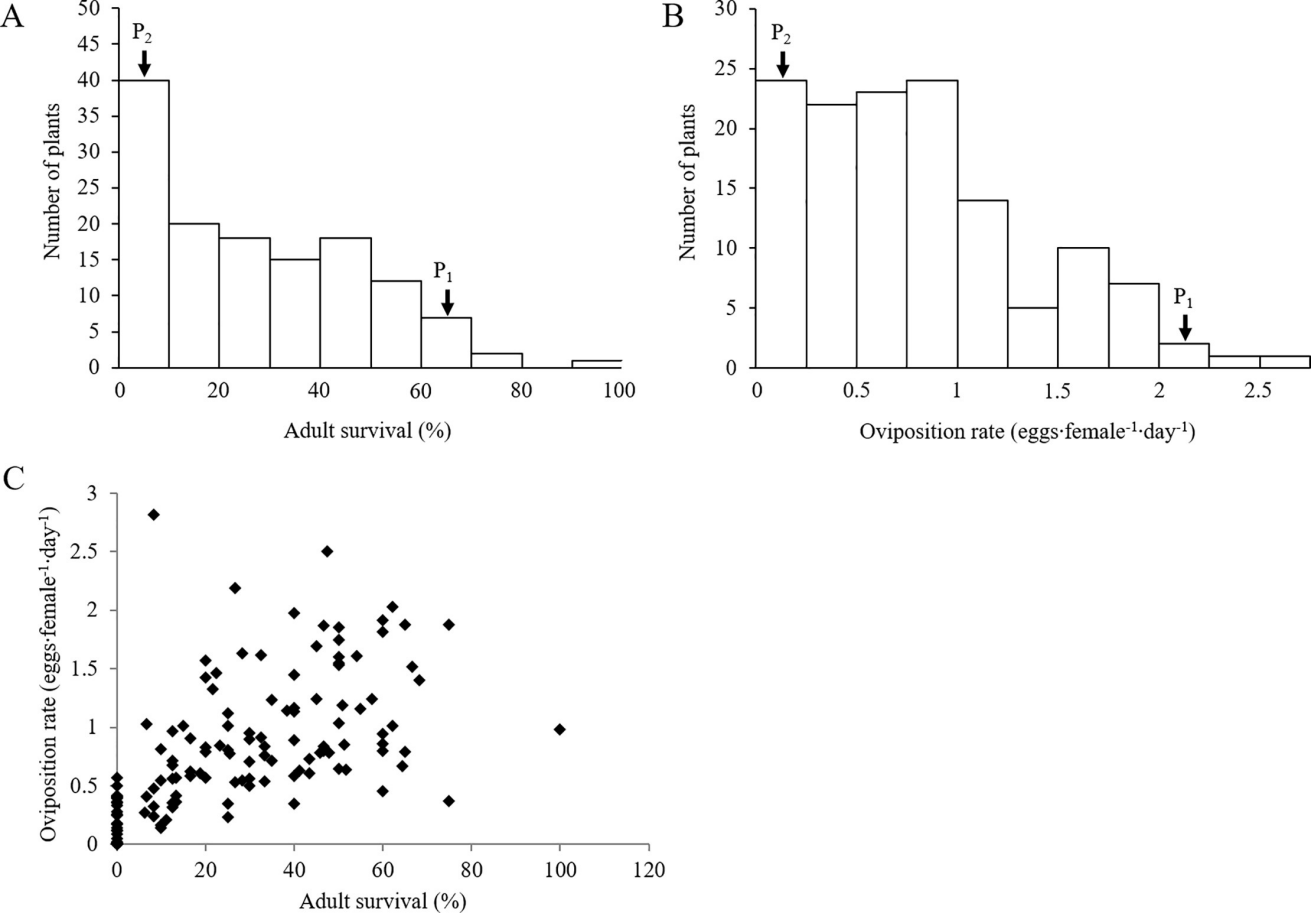
*Aleyrodes proletella* performance and relatedness between parameters on F_2_ plants derived from a cross between *Brassica oleracea* susceptible cv. Christmas Drumhead and resistant cv. Rivera. Histograms of (A) adult survival (%) and (B) oviposition rate (eggs·female^-1^·day^-1^) on leaves of F_2_ plants. P_1_: Christmas Drumhead; P_2_: Rivera. (C) Scatterplot of adult survival against oviposition rate. Each data point represents individual F_2_ plants.

To be able to identify genes that are involved in conferring resistance, we crossed Rivera with the susceptible cabbage variety Christmas Drumhead and developed an F_2_ population in order to perform a QTL mapping study. In a field experiment, whitefly performance was monitored on 12-weeks-old plants of the F_2_ population (179 individuals), the resistant Rivera and the susceptible Christmas Drumhead. In accordance with previous experiments [16,23,24], the parental lines showed pronounced differences in whitefly performance. On Rivera plants, all whiteflies had died after 1 week and only a few eggs were laid (oviposition rate: 0.01 ± 0.02 eggs·female^-1^·day^-1^). On Christmas Drumhead plants, 68% (± 25) of the whiteflies survived and they had laid 2.03 (± 0.49) eggs·female^-1^·day^-1^ during the 1-week infestation period. Whitefly survival on plants of the F_2_ population ranged from zero to 100% (Fig 5A) and oviposition rate on these plants ranged from 0 to 2.8 eggs∙female^-1^∙day^-1^ (Fig 5B and S5 Table). On some F_2_ plants whiteflies did not survive but were able to lay some eggs before dying. Overall, 38–41% of the plants were highly resistant (survival < 20% and/or oviposition rate < 0.5 eggs·female^-1^·day^-1^) among which 10% were completely resistant (no survival, no eggs; Fig 5). The broad-sense heritability (H^2^) was estimated at 0.43 for adult survival and 0.84 for oviposition rate. Across the F_2_ population, there was a significant correlation between adult survival and oviposition rate (*P* < 0.001, Pearson’s *r* = 0.69; Fig 5C).

### QTL mapping of whitefly resistance

**Fig 6:**
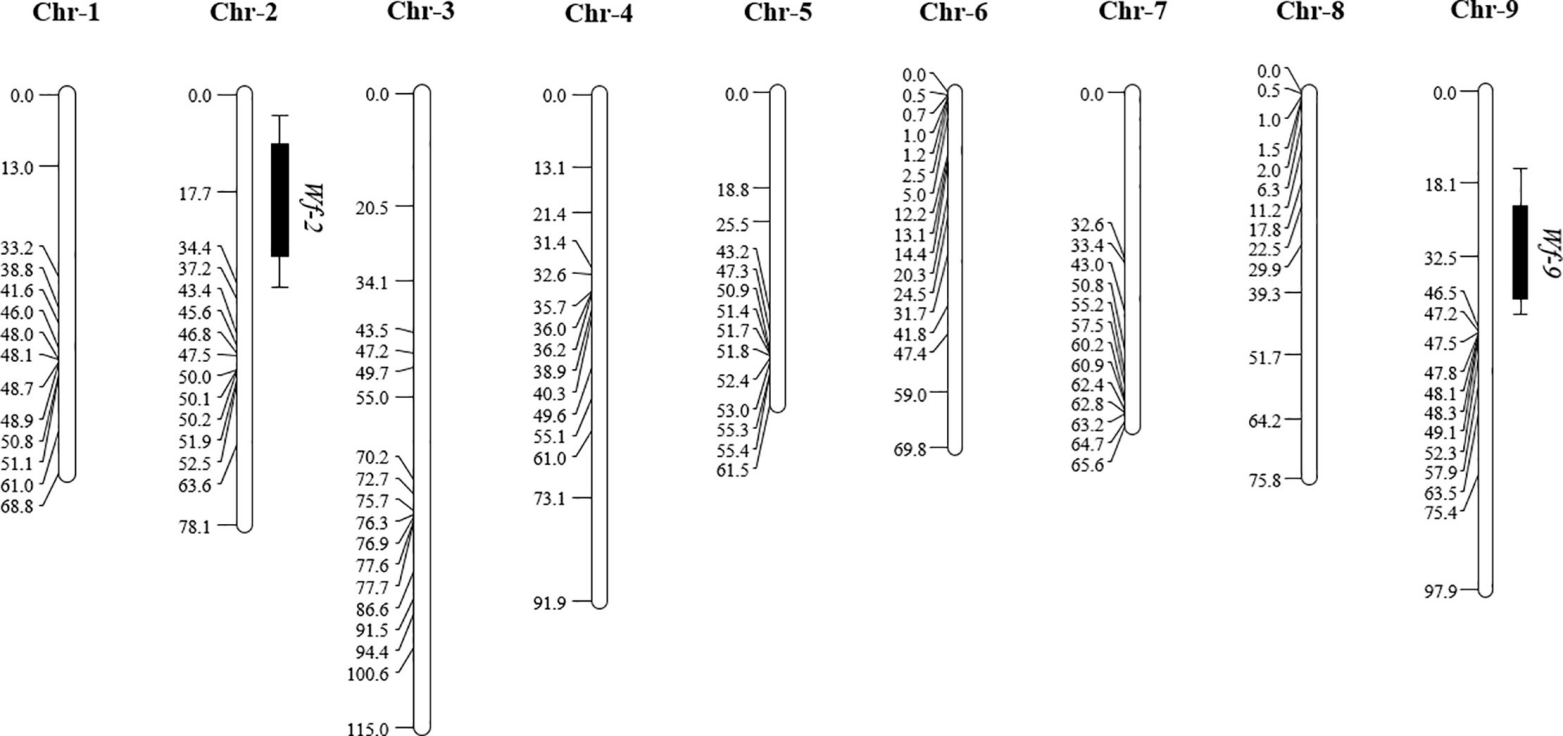
Genetic linkage maps of the 9 *Brassica oleracea* chromosomes. The map is based on 142 SNP markers segregating in the Christmas Drumhead x Rivera F_2_ population. Significant QTLs for whitefly oviposition rate (*Wf-2*) and adult survival (*Wf-9*) are shown on the right side of the chromosome bars. For each QTL the inner 1-LOD (wide line) and outer 2-LOD (thin line) intervals are specified. Genetic distances (cM) are shown on the left side of the chromosome bars. Chr, indicate chromosome.

A genetic linkage map was constructed covering all the 9 chromosomes of *B*. *oleracea* (Fig 6). The genetic map has a total length of 724 cM with an average marker interval of 5.6 cM. Distorted allele frequencies were only observed for markers on chromosome 7 towards Christmas Drumhead alleles. Interval mapping of the whitefly performance data identified 2 QTLs for both whitefly performance parameters individually (Fig 6 and Table 1): a QTL for oviposition rate on chromosome 2 (*Wf-2*), covering 32 cM and harboring about 2300 genes, and a QTL for adult survival on chromosome 9 (*Wf-9*), covering 29 cM and harboring about 1600 genes.

**Table 1:** QTLs for *A*. *proletella* performance identified using linkage mapping in the Christmas Drumhead x Rivera F_2_ population.

Trait	QTL	Chr.	SNP marker^a^	Position (cM)	GW LOD^b^	LOD^c^	R^2^ (%)^d^
Oviposition rate	*Wf-2*	2	C2_1BS859951405	17.7	3.5	3.9	13.3
Adult survival	*Wf-9*	9	C9_2BS2433972089	32.5	3.4	4.9	16.5

^a^The most significant marker

^b^GW LOD: Genome Wide LOD (Log10 (probability of linkage/probability of no linkage)) threshold

^c^LOD: LOD at most significant marker

^d^ R^2^, percentage of phenotypic variation explained by the QTL.

### Identification of candidate genes for resistance

Besides developing resistance, plants go through many other changes during their development. Therefore, the majority of the DEGs between young and older plants are most likely involved in general plant development processes and not related to resistance against the cabbage whitefly. To identify candidate genes for resistance, we looked for co-localization of DEGs in the QTL 2-LOD interval regions identified for whitefly performance on chromosome 2 and 9. In *Wf-2*, 35 DEGs showed higher expression levels in young plant while in *Wf-9* the number of DEGs with higher expression levels in young plants was 14 (S6 Table). The genes with higher levels of expression in young plants located in the QTL region are mainly orthologous to Arabidopsis genes involved in development and general plant processes such as photosynthesis. Twenty-two of the DEGs with higher expression levels in older plants were located in one of the identified QTLs, 13 in *Wf-2* (oviposition rate) and 9 in *Wf-9* (adult survival) (Table 2). Both QTLs harbor genes with higher expression levels in older plants that are orthologous to Arabidopsis genes that have been related to ABA signaling, including *AAO1* (Bo2g023330), *MYB96* (Bo9g014980) and *RD22* (Bog011300) [47,48].

**Table 2:** Identity and annotated function of genes within mapped QTL intervals that are also higher expressed in older, resistant plants.

*Brassica oleracea* gene ID	Arabidopsis orthologue	Annotated function	Fold change^a^	QTL
Bo2g002730	AT5G02790	Glutathione S-transferase family protein (GSTL3)	1.66	*Wf-2*
Bo2g002740	AT5G02800	Protein kinase family protein	1.84	*Wf-2*
Bo2g006660	AT5G04740	ACT domain-containing protein	4.14	*Wf-2*
Bo2g007200	AT5G06300	Cytokinin riboside 5'-monophosphate phosphoribohydrolase	2.17	*Wf-2*
Bo2g007260	AT5G06530	ABC transporter G family member 22	3.56	*Wf-2*
Bo2g008820	AT5G07920	Diacylglycerol kinase1 (DGK1)	1.74	*Wf-2*
Bo2g009180	AT5G08660	Hypothetical protein	1.68	*Wf-2*
Bo2g009640	AT5G09960	Hypothetical protein	4.13	*Wf-2*
Bo2g010640	AT5G11790	Protein N-MYC downregulated-like 2 (NDL2)	2.58	*Wf-2*
Bo2g010810	AT5G12080	Mechanosensitive channel of small conductance-like 10 (MSL10)	1.41	*Wf-2*
Bo2g018480	AT5G19900	Putative PRLI-interacting factor	1.88	*Wf-2*
Bo2g018630	AT5G20110	Dynein light chain type 1-like protein	1.83	*Wf-2*
Bo2g023330	AT5G20960	Aldehyde oxidase 1 (AAO1)	11.10	*Wf-2*
Bo9g024550	AT2G17500	Auxin efflux carrier-like protein	4.27	*Wf-9*
Bo9g011300	AT5G25610	Dehydration-responsive protein (RD22)	51.18	*Wf-9*
Bo9g010300	AT5G27240	DNAJ heat shock N-terminal domain-containing protein	1.30	*Wf-9*
Bo9g009850	AT5G49480	Ca2+-binding protein 1 (CP1)	7.46	*Wf-9*
Bo9g014830	AT5G62165	Protein agamous-like 42 (AGL42)	5.52	*Wf-9*
Bo9g014980	AT5G62470	Myb domain protein 96 (MYB96)	2.48	*Wf-9*
Bo9g016340	AT5G62960	Hypothetical protein	4.28	*Wf-9*
Bo9g018660	AT5G65380	Mate efflux domain-containing protein	1.59	*Wf-9*
Bo9g021820	AT5G66620	Protein DA1-related 6 (DAR6)	4.12	*Wf-9*

^a^ the factor by which a certain gene was more strongly expressed in older plants.

## Discussion

### Whitefly resistance as well as hormone and glucosinolate regulation changes during plant

Similar to Rivera, plant-age dependent resistance towards the cabbage whitefly has previously been identified in several cabbage varieties [16]. Here we show that the plant-age dependent resistance in Rivera develops very quickly, during a 2-week transition phase, with an intermediate phase in between. As the transition from susceptible to resistance occurs more or less together with the start of head formation, plants most likely start to change their root:shoot ratio around that time and consequently change resource allocation from growth to resistance [49].

Increased defense trait expression during plant development has previously been reported for constitutive accumulation of defensive compounds as well as for induced responses to insect attack [50,51]. Both these findings also hold for Rivera in its interaction with the cabbage whitefly. Genes related to hormone signaling and secondary metabolite biosynthesis were higher expressed in older resistant plants than in young susceptible plants, which was reflected in the constitutive levels of the phytohormone auxin and especially the aliphatic glucosinolates.Because auxin is an important player in plant developmental processes, higher levels in young than in older plants make sense in that perspective but does not rule out the possibility of auxin as a susceptibility factor in the interaction between the cabbage whitefly and Rivera. Glucosinolates have been shown to affect phloem-feeding insects, although this mainly holds for indole glucosinolates [9–11]. Upon whitefly infestation, regulation of ABA was contrasting for both plant ages of Rivera, i.e., reduced ABA levels in young plants, while older plants showed increased accumulation. A role for ABA in defense against caterpillars has been shown previously [4,6], but our results suggest a role for this hormone in resistance against phloem-feeding whiteflies as well.

Surprisingly, while JA has generally been considered as the major player in plant defense responses to insect herbivores [3], accumulation of this hormone was reduced in both plant ages upon whitefly infestation. This may be related to the hypothesis that phloem-feeding insects are able to manipulate the defenses of plants in order to establish a compatible interaction [52]. The silverleaf whitefly has been shown to induce SA in Arabidopsis, possibly to suppress effectual JA defenses [14]. Although we did not see an induction of SA in our study, it is very well possible that the suppression of JA signaling contributes to the susceptibility of young plants towards whitefly infestation. The whitefly resistance that develops during plant growth is in that case based on a different mechanism, possible regulated by ABA, that can overrule the JA-manipulated compatibility.

### Whitefly resistance is a dominantly inherited, polygenic trait

To characterize the genetics of whitefly resistance in Rivera, we conducted a QTL mapping study using a mapping population derived from a cross between susceptible *B*. *oleracea* Christmas Drumhead [23] and Rivera. The F_2_ population showed a skewed distribution towards resistance, indicating a (partially) dominant inheritance of the resistance. The lower heritability for adult survival (0.43) compared with that for oviposition rate (0.84) suggests that adult survival is somewhat influential by environmental factors but that once the females establish a suitable feeding site, their performance is mainly influenced by genetic factors. This is consistent with previous findings showing that the resistance in Rivera is highly robust, i.e., reproducible in field experiments across different years [16,23,24]. The QTL analysis revealed 2 QTLs, one for adult survival and one for oviposition rate, both explaining a small percentage of the phenotyping variation and with LOD scores just above the threshold. These results suggest that whitefly resistance in Rivera is based on multiple genes, each contributing a small part to the overall resistance. A similar low fraction of explained variance was also observed in several other studies on host plant resistance towards phloem-feeding insects in which multiple QTLs were identified. For example, several minor QTLs were detected in tomato for adult survival and oviposition rate of the silverleaf whitefly [53]. In melon, 2 minor QTLs were detected for the number of silverleaf whitefly progenies, explaining 14–18% of the genetic variation [18]. It is likely that additional QTLs for whitefly performance in cabbage are present and may be detected by increasing the mapping population size, the number of polymorphic markers or by phenotyping F_3_ lines.

Despite the correlation between the whitefly performance parameters, the identified QTLs were located at different positions, namely on chromosome 2 (oviposition rate) and 9 (adult survival). Similar results were also found for SNPs associated with cabbage whitefly performance in Arabidopsis [54], suggesting the existence of multiple defense mechanisms that each have a different effect on whitefly performance. A possible mechanism involves lethal traits that cause whiteflies to die very quickly, i.e., within several days, and prevent oviposition. As oviposition generally occurs during feeding, such a lethal trait most likely interferes with phloem sap ingestion, which has previously been shown for whiteflies feeding in on Rivera leaves of older plants [23]. Several plants on which (almost) all whiteflies died during the course of infestation did show a significant number of eggs suggesting that the whiteflies were able to feed for a short period before dying. This suggests the existence of another mechanism involving traits that negatively affect whitefly performance but do not cause a sudden death, a phenomenon that has been shown for the silverleaf whitefly on tomato [21] and Arabidopsis [54]. More detailed phenotyping, such as daily monitoring of adult survival and egg numbers, is needed to distinguish the mechanisms behind such different defensive traits.

### ABA signaling possibly plays a role in the regulation of resistance

The value of combining genetic mapping with transcriptome profiling to narrow down the number of candidate genes has been shown for several complex traits of plants [55–58]. Such an approach has also been used in *Nicotiana benthamiana* to identify genes involved in the production of saponins, defensive compounds that can deter insects [59]. Here, we used the combination approach to reduce the number of candidate genes for whitefly resistance. Although the identified QTL regions are large, the expression patterns of the genes within these regions allow the selection of a manageable number of candidates. Several of the identified candidate genes, i.e., differentially expressed and located in the QTL region for oviposition rate or adult survival, are orthologous to Arabidopsis genes that have been shown to play a role in ABA signaling. In *Wf-2*, *Bo2g023330* is orthologous to Arabidopsis *AAO1*, a gene involved in catalyzing the final step of the ABA biosynthesis pathway [47]. In the same QTL, *Bo2g007260* is orthologous to Arabidopsis *ABCG22* that functions in ABA signaling and biosynthesis [60] and *Bo2g006660* is orthologous to Arabidopsis *At5g04740*, an ABA responsive gene [61]. In *Wf-9*, the orthologue of *Bo9g014980* in Arabidopsis is *MYB96*, a critical component of ABA signaling [48]. Additionally, MYB96 can also mediate ABA signals to regulate drought resistance via *RD22* [48], of which the *B*. *oleracea* orthologue (*Bo9g011300*) was also higher expressed in older plants and located in *Wf-9*.

The plant hormone ABA functions in many plant developmental processes but can also play an important regulatory role in response to abiotic and biotic stress [62]. Drought, for example, induces ABA biosynthesis to initiate stomatal closure that does not only prevent further water loss but can also serve as a pre-invasive defense mechanism against pathogens [63]. However, only a limited number of studies have suggested a role for ABA in plant defense against insects [4,6,63,64]. The identification of genes related to ABA signaling as candidates for whitefly resistance together with the whitefly-induced increase of ABA levels in older plants suggests that ABA may also play an important role in plant defense against the cabbage whitefly.

## Supporting information

S1 Table Performance of *Aleyrodes proletella* on young and older plants of *Brassica oleracea* variety Rivera used for RNA-seq analysis. (XLSX)

S2 Table List of age-dependent differentially expressed genes between young (6-weeks-old) and older (13-weeks-old) Rivera plants. (XLSX)

S3 Table List of significantly enriched Gene Ontology processes for DEGs with higher levels of expression in young, susceptible plants. (XLSX)

S4 Table List of significantly enriched Gene Ontology processes for DEGs with higher levels of expression in older, resistant plants. (XLSX)

S5 Table Whitefly performance on plant of an F_2_ population derived from a cross between susceptible cv. Christmas Drumhead and resistant cv. Rivera. (XLSX)

S6 Table Identity and annotated function of genes within mapped QTL intervals that are also higher expressed in young, susceptible plants. (XLSX)

## References

[1] Broekgaarden C, Snoeren TJ, Dicke M, Vosman B. Exploiting natural variation to identify insect-resistance genes. Plant Biotech J. 2011; 9: 819–825. 10.1111/j.1467-7652.2011.00635.x 21679292

[2] Pappas ML, Broekgaarden C, Broufas GD, Kant MR, Messelink GJ, Steppuhn A, et al Induced plant defences in biological control of arthropod pests: a double-edged sword. Pest Manag Sci. 2017; 73: 1780–1788. 10.1002/ps.4587 28387028 PMC5575458

[3] Bodenhausen N, Reymond P. Signaling pathways controlling induced resistance to insect herbivores in Arabidopsis. Mol Plant Microbe Interact. 2007; 20: 1406–1420. 10.1094/MPMI-20-11-1406 17977152

[4] Verhage A, Vlaardingerbroek I, Raaijmakers C, van Dam NM, Dicke M, van Wees SCM, Pieterse CMJ. Rewiring of the jasmonate signaling pathway in Arabidopsis during insect herbivory. Front Plant Sci. 2011; 2: 47 10.3389/fpls.2011.00047 22645537 PMC3355780

[5] Broekgaarden C, Caarls L, Vos IA, Pieterse CMJ, van Wees SCM. Ethylene: traffic controller on hormonal crossroads to defense. Plant Physiol. 2015; 169: 2371–2379. 10.1104/pp.15.01020 26482888 PMC4677896

[6] Vos IA, Verhage A, Schuurink RC, Watt LG, Pieterse CMJ, van Wees SCM. Onset of herbivore-induced resistance in systemic tissue primed for jasmonate-dependent defenses is activated by abscisic acid. Front Plant Sci. 2013; 4: 539 10.3389/fpls.2013.00539 24416038 PMC3874679

[7] Pieterse CMJ, van der Does D, Zamioudis C, Leon-Reyes A, van Wees SCM. Hormonal modulation of plant immunity. Ann Rev Cell Dev Biol. 2012; 28: 489–521. 22559264 10.1146/annurev-cellbio-092910-154055

[8] Schweizer F, Fernández-Calvo P, Zander M, Diez-Diaz M, Fonseca S, Glauser G, et al Arabidopsis basic helix-loop-helix transcription factors MYC2, MYC3, and MYC4 regulate glucosinolate biosynthesis, insect performance, and feeding behavior. Plant Cell. 2013; 25: 3117–3132. 10.1105/tpc.113.115139 23943862 PMC3784603

[9] Züst T, Agrawal AA. Mechanisms and evolution of plant resistance to aphids. Nature Plants. 2016; 2: 15206 10.1038/nplants.2015.206 27250753

[10] Elbaz M, Halon E, Malka O, Malitsky S, Blum E, Aharoni A, Morin S. Asymmetric adaptation in indolic and aliphatic glucosinolates in the B and Q sibling species of *Bemisia tabaci* (Hemptera: Aleyrodidae). Mol Ecol. 2012; 21: 4533–4546. 10.1111/j.1365-294X.2012.05713.x 22849567

[11] Markovich O, Kafle D, Elbaz M, Malitsky S, Aharoni A, Schwarzkopf A, et al *Arabidopsis thaliana* plants with different levels of aliphatic- and indolyl-glucosinolates affect host selection and performance of *Bemisia tabaci*. J Chem Ecol. 2013; 39: 1361–1372. 10.1007/s10886-013-0358-0 24190022

[12] Wang XW, Li P, Liu SS. Whitefly interactions with plants. Curr Opin Insect Sci. 2017; 19: 70–75. 10.1016/j.cois.2017.02.001 28521945

[13] Li J, Zhu L, Hull JJ, Liang S, Daniell H, Jin S, Zhang X. Transcriptome analysis reveals a comprehensive insect resistance response mechanism in cotton to infestation by the phloem feeding insect *Bemisia tabaci* (whitefly). Plant Biotech J. 2016; 14: 1956–1975. 10.1111/pbi.12554 PMC5042180 26923339

[14] Zarate SI, Kempema LA, Walling LL. Silverleaf whitefly induces salicylic acid defenses and suppresses effectual jasmonic acid defenses. Plant Physiol. 2007; 143: 866–875. 10.1104/pp.106.090035 17189328 PMC1803729

[15] Bleeker PM. Diergaarde PJ, Ament K, Guerra J, Weidner M, Schütz S, et al The role of specific tomato volatiles in tomato-whitefly interaction. Plant Physiol 2009; 151: 925–935 10.1104/pp.109.142661 19692533 PMC2754627

[16] Pelgrom KTB, Broekgaarden C, Voorrips RE, Bas N, Visser RGF, Vosman B. Host plant resistance towards the cabbage whitefly in *Brassica oleracea* and its wild relatives. Euphyt. 2015; 202: 297–306.

[17] Vosman B, van ‘t Westeinde WPC, Henken B, van Eekelen HDLM, de Vos RCH, Voorrips RE. Broad spectrum insect resistance and metabolites in close relatives of the cultivated tomato. Euphyt. 2018; 214: 46. 10.1007/s10681-018-2124-4 PMC6445503 31007274

[18] Boissot N. Thomas S, Sauvion N, Marchal C, Pavis C, Dogimont C. Mapping and validation of QTLs for resistance to aphids and whiteflies in melon. Theor Appl Genet. 2010; 121: 9–20. 10.1007/s00122-010-1287-8 20180095

[19] Perez-Sackett PT, Cianzio SR, Kara PC, Aviles M, Palmer RG. QTL mapping of whitefly resistance in soybean. J Crop Imp. 2011; 25: 134–150.

[20] Firdaus S, van Heusden AW, Hidayati N, Supena EDJ, Mumm R, de Vos RCH, et al Identification and QTL mapping of whitefly resistance components in *Solanum galapagense*. Theor Appl Genet. 2013; 126: 1487–1501. 10.1007/s00122-013-2067-z 23440381

[21] Lucatti AF, Meijer-Dekens FRG, Mumm R, Visser RGF, Vosman B, van Heusden S. Normal adult survival but reduced *Bemisia tabaci* oviposition rate on tomato lines carrying an introgression from *S*. *habrochaites*. BMC Genet. 2014; 15: 142 10.1186/s12863-014-0142-3 25539894 PMC4301655

[22] Nombela G, Williamsom VM, Muñiz M. The root-know nematode resistance gene *Mi-1*.*2* of tomato is responsible for resistance against the whitefly *Bemisia tabaci*. Mol Plant Microbe Int. 2003; 16: 645–649. 10.1094/MPMI.2003.16.7.645 12848430

[23] Broekgaarden C, Riviere P, Steenhuis G, del sol Cuenca M, Kos M, Vosman B. Phloem-specific resistance in *Brassica oleracea* against the whitefly *Aleyrodes proletella*. Entomol Exp Appl. 2012; 142: 153–164.

[24] Broekgaarden C, Poelman EH, Voorrips RE, Dicke M, Vosman B. Intraspecific variation in herbivore community composition and transcriptional profiles in field-grown *Brassica oleracea* cultivars. J Exp Bot. 2009; 61: 807–819. 10.1093/jxb/erp347 19934173 PMC2814112

[25] Hickman RJ, van Verk MC, van Dijken AJH, Pereira Mendes M, Vroegop-Vos IA, Caarls L, et al Architecture and dynamics of the jasmonic acid gene regulatory network. Plant Cell. 2017; 29: 2086–2105. 10.1105/tpc.16.00958 28827376 PMC5635973

[26] Parkin IA, Koh C, Tang H, Robinson S, Kagale S, Clarke WE, et al Transcriptome and methylome profiling reveals relics of genome dominance in the mesopolyploid *Brassica oleracea*. Gen Biol. 2014; 15: R77. 10.1186/gb-2014-15-6-r77 PMC4097860 24916971

[27] Bray NL, Pimentel H, Melsted P, Pachter L. Near-optimal probabilistic RNA-seq quantification. Nat Biotechnol. 2016; 34: 525–527. 10.1038/nbt.3519 27043002

[28] Robinson MD, McCarthy DJ, Smyth GK. edgeR: a Bioconductor package for differential expression analysis of digital gene expression data. Bioinformat. 2010; 26: 139–140. 10.1093/bioinformatics/btp616 PMC2796818 19910308

[29] Thomas PD, Campbell MJ, Kejariwal A, Mi H, Karlak B, Daverman R, et al PANTHER: a library of protein families and subfamilies indexed by function. Genome Res. 2003; 13: 2129–2141. 10.1101/gr.772403 12952881 PMC403709

[30] Papadopoulou GV, Maedicke A, Grosser K, van Dam NM, Martínez-Medina A. Defence signaling marker gene responses to hormonal elicitation differ between roots and shoots. AoB Plants. 2018; 10: ply031. 10.1093/aobpla/ply031 PMC6007416 29977487

[31] Grosser K, van Dam NM. A straightforward method for glucosinolate extraction and analysis with high-pressure liquid chromatography (HPLC). J Vis Exp. 2017; 121: e55425. 10.3791/55425 PMC5409297 28362416

[32] Fulton TM, Chunwongse J, Tanksley SD. Microprep protocol for extaction of DNA from tomato and other herbaceous plants. Plant Mol Biol Rep. 1995; 13: 207–209.

[33] Magoč T, Salzberg SL. FLASH: Fast length adjustment of short reads to improve genome assemblies. Bioinformatics. 2011; 27: 2957–2963. 10.1093/bioinformatics/btr507 21903629 PMC3198573

[34] Smeds L, Künster A. ConDeTri–a content dependent read trimmer for Illumina data. PloS one. 2011; 6: e26314 10.1371/journal.pone.0026314 22039460 PMC3198461

[35] Li R, Zhu H, Ruan J, Qian W, Fang X, Shi Z, et al De novo assembly of human genomes with massively parallel short read sequencing. Gen Res. 2010; 20: 265–272. 10.1101/gr.097261.109 PMC2813482 20019144

[36] Langmead B, Salzberg SL. Fast gapped-read alignment with Bowtie 2. Nat Methods. 2012; 9: 357–359. 10.1038/nmeth.1923 22388286 PMC3322381

[37] Nijveen H, van Kaauwen M, Esselink DG, Hoegen B, Vosman B. QualitySNPng: a user-friendly SNP detection and visualization tool. Nucl Acid Res. 2013; 41: W587–W590. 10.1093/nar/gkt333 PMC3692117 23632165

[38] Tang J, Vosman B, Voorrips RE, van der Linden CG, Leunissen J. QualitySNP: a pipeline for detecting single nucleotide polymorphisms and insertions/deletions in EST data from diploid and polyploidy species. BMC Bioinformat. 2006; 7: 438. 10.1186/1471-2105-7-438 PMC1618865 17029635

[39] Cheng F, Liu S, Wu J, Fang L, Sun S, Liu B, Li P, Hua W, Wang X. BRAD, the genetics and genomics database for Brassica plants. BMC Plant Biol. 2011; 11:136 10.1186/1471-2229-11-136 21995777 PMC3213011

[40] Van Ooijen JW. Joinmap 4, software for the calculation of genetic linkage maps in experimental populations. Kyazma BV, Wageningen, the Netherlands 2006.

[41] Voorips RE. MapChart: software for the graphical representation of linkage maps and QTLs. J Her. 2002; 93: 77–78. 10.1093/jhered/93.1.77 12011185

[42] Van Ooijen JW. MapQTL6, software for the mapping of quantitative trait loci in experimental populations of diploid species. Kyazma BV, Wageningen, the Netherlands 2009.

[43] De Moel Cp, Zwanepol S, Everaarts A, Alblas J, Hoek H. Teelt van sluitkool. PAGV, Lelystad, the Netherlands 1996.

[44] Kazan K, Manners JM. MYC2: the master in action. Mol Plant. 2013; 6: 686–703. 10.1093/mp/sss128 23142764

[45] Fernández-Calvo P, Chini A, Fernández-Barbero G, Chico JM, Gimenez-Ibanez S, Geerinck J, et al The Arabidopsis bHLH transcription factors MYC3 and MYC4 are targets of JAZ repressors and act additively with MYC2 in the activation of jasmonate responses. The Plant Cell. 2011; 23: 701–715. 10.1105/tpc.110.080788 21335373 PMC3077776

[46] Dixon DP, Hawkins T, Hussey PJ, Edwards R. Enzyme activities and subcellular localization of members of the Arabidopsis glutathione transferase superfamily. J Exp Bot. 2009; 60: 1207–1218. 10.1093/jxb/ern365 19174456 PMC2657551

[47] Vishwakarma K, Upadhyay N, Kumar N, Yadav G, Singh J, Mishra RK, et al Abscisic acid signaling and abiotic stress tolerance in plants: a review on current knowledge and future prospects. Front Plant Sci. 2017; 8: 161 10.3389/fpls.2017.00161 28265276 PMC5316533

[48] Seo PJ, Xiang F, Qiao M, Park JY, Lee YN, Kim SG, et al The MYB96 transcription factor mediates abscisic acid signaling during drought stress response in Arabidopsis. Plant Physiol. 2009; 151: 275–289. 10.1104/pp.109.144220 19625633 PMC2735973

[49] Boege K, Marquis RJ. Facing herbivory as you grow up: the ontogeny of resistance in plants. Trends Ecol Evol. 2005; 20: 441–448. 10.1016/j.tree.2005.05.001 16701415

[50] Barton KE, Koricheva J. The ontogeny of plant defense and herbivory: characterization general patterns using meta-analysis. Amer Nat. 2010; 175: 481–493. 20170370 10.1086/650722

[51] Mao Y-B, Liu Y-Q, Chen D-Y, Chen F-Y, Fang X, Hong G-J, et al Jasmonate response decay and defense metabolite accumulation contributes to age-related dynamics of plant insect resistance. Nature Comm. 2015; 8: 13925. 10.1038/ncomms13925 PMC5233801 28067238

[52] Walling LL. Avoiding effective defenses: strategies employed by phloem-feeding insects. Plant Physiol. 2008; 146: 859–866. 10.1104/pp.107.113142 18316641 PMC2259051

[53] Van den Oever-van den Elsen F, Lucatti AF, van Heusden S, Broekgaarden C, Mumm R, Dicke M, Vosman B. Quantitative resistance against *Bemisia tabaci* in *Solanum pennellii*: genetics and metabolomics. J Int Plant Biol. 2016; 58: 397–412. 10.1111/jipb.12449 26576823

[54] Broekgaarden C, Bucher J, Bac-Molenaar J, Keurentjes JJB, Kruijer W, Voorrips RE, Vosman B. Novel genes affecting the interaction between the cabage whitefly and *Arabidopsis* uncovered by genome-wide association mapping. PloS one. 2015; 10: e0145124 10.1371/journal.pone.0145124 26699853 PMC4689410

[55] Marino R, Ponnaiah M, Krajewski P, Frova C, Gianfranceschi L, Pè ME, Sari-Gorla M. Addressing drought tolerance in maize by transcriptional profiling and mapping. Mol Genet Gen. 2009; 281: 163–179. 10.1007/s00438-008-0401-y 19018570

[56] Pandit A, Rai V, Bal S, Sinha S, Kumar V, Chauhan M, et al Combining QTL mapping and transcriptome profiling of bulked RILs for identification of functional polymorphism for salt tolerance genes in rice (*Oryza sativa* L.). Mol Genet Genom. 2010; 284: 121–136. 10.1007/s00438-010-0551-6 20602115

[57] Gelli M, Mitchell SE, Liu K, Clemente TE, Weeks DP, Zhang C, et al Mapping QTLs and association of differentially expressed gene transcripts for multiple agronomic traits under different nitrogen levels in sorghum. BMC Plant Biol. 2016; 16: 16 10.1186/s12870-015-0696-x 26759170 PMC4710988

[58] Zhang D, Zhang H, Chu S, Li H, Chi Y, Triebwasser-Freese D, et al Integrating QTL mapping and transcriptomics identifies candidate genes underlying QTLs associated with soybean tolerance to low-phosphorus stress. Plant Mol Biol. 2017; 93: 137–150. 10.1007/s11103-016-0552-x 27815671

[59] Khakimov B, Kuzina V, Erthmann PØ, Fukushima EO, Augustin JM, Olsen CE, et al Identification and genome organization of saponin pathway genes from a wild crucifer, and their use for transient production of saponins in *Nicotiana benthamiana*. Plant J. 2015; 84: 478–490. 10.1111/tpj.13012 26333142

[60] Kuromori T, Sugimoto E, Shinozaki K. Arabidopsis mutants of *AtABCG22*, an ABC transporter gene, increase water transpiration and drought susceptibility. Plant J. 2011; 67: 885–894. 10.1111/j.1365-313X.2011.04641.x 21575091

[61] Böhmer M, Schroeder JI. Quantitative transcriptomic analysis of abscisic acid-induced and reactive oxygen species-dependent expression changes and proteomic profiling in Arabidopsis suspension cells. Plant J. 2011; 67: 105–118. 10.1111/j.1365-313X.2011.04579.x 21426425 PMC3125488

[62] Lee SG, Luan S. ABA signal transduction at the crossroad of biotic and abiotic stress responses. Plant Cell Environ. 2011; 35: 53–60. 10.1111/j.1365-3040.2011.02426.x 21923759

[63] Thaler JS, Bostock RM. Interactions between abscisic acid mediated responses and plant resistance to pathogens and insects. Ecol. 2004; 85: 48–58.

[64] Pérez-Hedo M, Urbaneja-Bernat P, Jaques JA, Flors V, Urbaneja A. Defensive plant responses induced by *Mesidiocoris tenuis* (Hemiptera: Miridae) on tomato plants. J Pest Sci. 2015; 88: 543–554.

